# A Systematic Review of the Impact of Invasive Alien Plants on Forest Regeneration in European Temperate Forests

**DOI:** 10.3389/fpls.2020.524969

**Published:** 2020-09-03

**Authors:** Magdalena Langmaier, Katharina Lapin

**Affiliations:** ^1^Austrian Federal Research Centre for Forests, Natural Hazards and Landscape, Vienna, Austria; ^2^Institute of Silviculture, University of Natural Resources and Life Sciences Vienna, Vienna, Austria

**Keywords:** biological invasion, exotic plants, forest dynamics, ground vegetation, invasive species management, neophytes, rejuvenation, tree seedlings

## Abstract

Invasive alien species (IAS) are one of the major threats to global and local biodiversity. In forest ecosystems, the threats caused by IAS include hybridization, transmission of diseases and species competition. This review sets out to analyze the impact of alien plant species on forest regeneration, which we consider to be one of the key stages in tree ecology for the survival of forest ecosystems in the future. The focus of the study is directly relevant to practitioners, forest managers and the conservation management of forests. With this systematic review, we aim to provide an overview of 48 research studies reporting on the impact and/or management of IAS in European temperate forests. We followed a multi-step protocol for compiling the publications for the literature review, with nine search queries producing a total of 3,825 hits. After several reduction rounds, we ended up with a grand total of 48 papers. We identified 53 vascular plant species having a negative influence on forest regeneration in Central European forests. In total, 21 tree species are reported to be impacted by IAS in 24 studies. The results of the review synthesis show that five impact mechanisms affect the regeneration success of native tree species: competition for resources, chemical impact on regeneration, physical impact on regeneration, structural impact on regeneration and indirect impact through interaction with other species. We identified in our synthesis management measures that have been recommended for application at different stages of biological invasions. The associated costs and required resources of management measures are under-reported or not accessible by reviewing the scientific literature. We can thus conclude that it is very import to improve the links between science and practical forest management. We expect that this review will provide direction for invasive plant species research and management aimed at protecting biodiversity in European temperate forest ecosystems.

## Introduction

### Background

Invasive alien or non-native plant species (hereinafter: IAS *sensu* Pyšek et al., 2004) are one of the major threats to global and local biodiversity (IUCN Council, 2000). In addition, biological invasions have negative socio-economic and human health impacts (Howe and Smallwood, 1982; Villamagna and Murphy, 2010; Shackleton et al., 2018; Potgieter et al., 2019; Roy et al., 2019). The rapid spread of few IAS in forests was mediated by the positive socio-economic effects of potentially invasive plant species in urban and rural areas (Kowarik, 2010; Vaz et al., 2017; Vaz et al., 2018; Castro-Díez et al., 2019). Therefore, several policies, risk assessments and legislations are now in place to regulate the spread of IAS in forest ecosystems (Heywood and Brunel, 2009; Brundu and Richardson, 2016; Pötzelsberger et al., 2020). The threats caused by IAS in forest ecosystems include hybridization, transmission of diseases and species competition (Knapp and Canham, 2000; Ashton et al., 2005; Vilà et al., 2011; Medvecká et al., 2018). The establishment and spread of alien plants in temperate forest ecosystems have not been widely considered until recently (Essl et al., 2011; Wagner et al., 2017), and European temperate broadleaf and mixed forests in particular were previously considered less affected than other landcover types (Chytrý et al., 2009; Martin et al., 2009). However, an increasing number of studies now indicate that temperate forests as well as other forest biomes might not be as resilient as previously assumed (McEwan et al., 2009; Dreiss and Volin, 2013). In boreal forests, for instance, an ongoing invasion of alien plants was detected, despite the relatively low levels of anthropogenic disturbance (Sanderson et al., 2012).

A key component of biological invasions are human activities that modulate the introduction and spread of alien species, and a large number of human activities supporting the spread of alien plant species have been identified (Richardson et al., 2000; Richardson et al., 2007). Clearing, for example, may change light conditions and resource availability in ways that favour alien plant species. Furthermore, the construction of forest roads can promote the spread of alien plant seeds and specimens through the movement of contaminated soil and construction material (Joly et al., 2011; Muscolo et al., 2014; Kerri et al., 2016). Since the forest road system in European temperate forests is well-developed, the risk of forest roads functioning as pathways for invasion should not be underestimated (Woziwoda et al., 2018).

Besides human-mediated disturbances, natural disturbances are also increasing due to climate change. Events such as storms, flooding and forest fires provide alien plants with opportunities to invade temperate forests (Hobbs and Huenneke, 1992; Brooks et al., 2004; Lake and Leishman, 2004). The spread of IAS driven by disturbances is frequently linked to poor individual performance and reduced species diversity, which lead to irreversible changes in the species composition of understory vegetation (Sjöman et al., 2016; Seidl et al., 2017; Navarro et al., 2018). For example, a proliferation of shade-tolerant alien plant species such as *Impatiens parviflora* has been observed at many sites in European temperate broadleaf forests (Chmura and Sierka, 2007; Hejda, 2012; Jarčuška et al., 2016; Lapin et al., 2019). These changes in the species composition of understory may impact the competition between IAS and native species and affect site conditions. We argue that alien plant species may be influencing the successful regeneration of tree seedlings.

Forest managers distinguish between two types of regeneration (also referred to as rejuvenation), meaning the process by which forests are renewed: natural and artificial regeneration. Natural regeneration is based on recruitment deriving from seeds (seedlings) without human assistance, while artificial regeneration implies the planting of seedlings or the sowing of seeds. The regeneration process of forest trees occurs in different phases: a seedling phase, a juvenile intensive height growth phase and a maturity phase (Borghetti and Giannini, 2009). The success of both natural and artificial regeneration in European temperate forests is affected by a large number of biotic and abiotic factors. Gap dynamics, light intensity, soil humidity, climate-induced disturbance regimes as well as soil properties and the composition of the regeneration layer all modulate regeneration (Emborg, 1998; Svoboda et al., 2010; Muscolo et al., 2014; Tinya et al., 2019). Furthermore, the impact of deer browsing on tree regeneration is a common and widely discussed problem in many European temperate and boreal forest types (Ammer, 1996; Barančeková et al., 2007).

Besides the above mentioned factors, however, different phases of the regeneration process are reportedly also affected by above-ground and below-ground competition between tree seedlings and plant species of the understorey herbaceous vegetation cover (Davis et al., 1998; Ricard et al., 2003; Booth, 2004; Royo and Carson, 2008; De Lombaerde et al., 2019). De Lombaerde et al. (2019) argue that manipulation of the overstorey cover (also tree layer or canopy cover) to reduce its abundance and shade-casting ability can increase tree seedling competitiveness. However, stress factors during the regeneration phase of seedling establishment are particularly impactful in regard to the renewing of forest ecosystems (Terwei et al., 2013). In managed forests, the regeneration is following a series of anthropogenic disturbances due to silvicultural measures, such as harvesting and soil preparation, in addition to the above mentioned biotic and abiotic factors. During plantation establishment or natural forest regeneration, forest ecosystems may be impacted especially heavily by alien plant invasions due to the increase in light and nutrient availability that intensifies competition for limited sources. Furthermore, understanding the impact mechanisms of alien plant species during the critical stage of forest regeneration has important implications for invasive species management and forest management in general.

### Objective and Aim

This review sets out to analyze the impact of alien plant species on forest regeneration, which we consider to be one of the key stages in tree ecology for the survival of forest ecosystems in the future. The focus of this study is directly relevant to practitioners, forest managers and the conservation management of forests.

With this review, we aim to provide an overview of 48 research studies reporting on the impact and/or management of IAS in European temperate forests. This information has hitherto not been systematically collated and analyzed—a process required to identify research lacunae and delineate the current state of the art. Against this background, we addressed the following research questions:

1. Which IAS have a negative influence on forest regeneration in Central European forests?
2. The regeneration of which specific tree species in Central European forests is threatened by IAS?
3. Which mechanisms and features of IAS have an impact on regeneration?
4. Which management measures and considerations can be employed to counter the impact of IAS on regeneration?

## Methods

### Ethics Statement

This paper does not involve field studies or primary data—it is purely an analysis of information in existing peer-reviewed studies (**Figure 2**).

### Study Area

**Figure 1:**
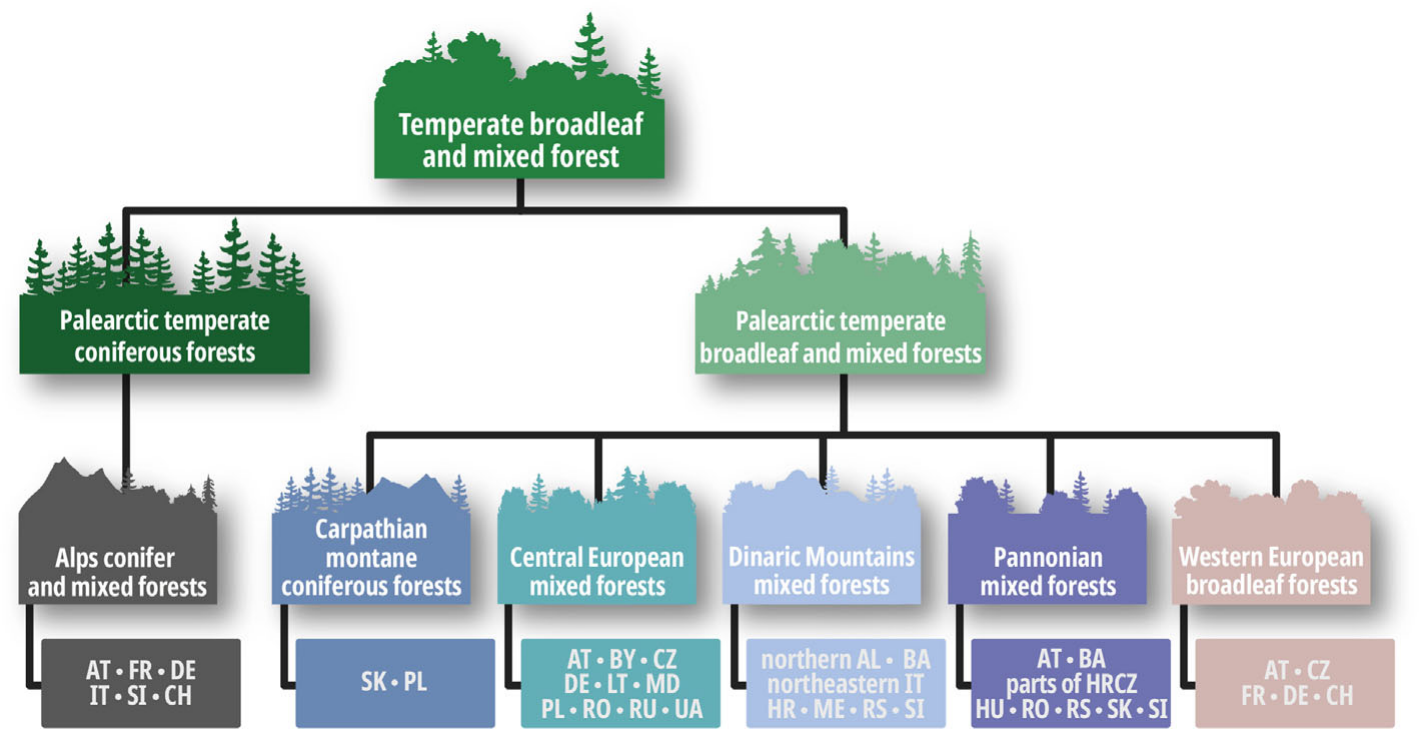
Overview of the ecological regions and countries from the Digital Map of European Ecological Regions appearing in the study.

To address our research questions, we decided to take a closer look at Central Europe, establishing a clear structure by using the terrestrial ecoregions classification according to the WWF (WWF, 2020). The terrestrial ecoregions include 14 major habitat types ranging from the wettest of forest types to the driest and hottest desert conditions. Each of these 14 major habitat types has a wide range of ecoregion categories, which are in turn divided into subtypes. For Europe, this subtype classification is equivalent to the Digital Map of European Ecological Regions (hereinafter DMEER) (Bohn et al., 2003) as shown in **Figure 1**. The major habitat for Central Europe are the “temperate broadleaf and mixed forests” with their two habitat subtypes “palearctic temperate coniferous forests” and “palearctic temperate broadleaf and mixed forests”. The typical characteristic of the “temperate broadleaf and mixed forests” is a forest structure consisting of four layers: The main layer is a canopy with dominant mature species, which is accompanied by a second layer of trees, a shrub layer, and a grass and herb layer. Within the habitat subtype “palearctic temperate coniferous forests”, we reviewed studies pertaining to countries belonging to the subdivision “Alps conifer and mixed forests”. Within the habitat subtype “palearctic temperate broadleaf and mixed forests”, we reviewed studies pertaining to countries belonging to the subdivisions “Dinaric Mountains mixed forests”, “Pannonian mixed forests”, “Central European mixed forests”, and “Western European broadleaf forests”. **Figure 3** provides an overview of the specific countries covered in the reviewed studies.

### Search Query and Criteria

**Figure 2:**
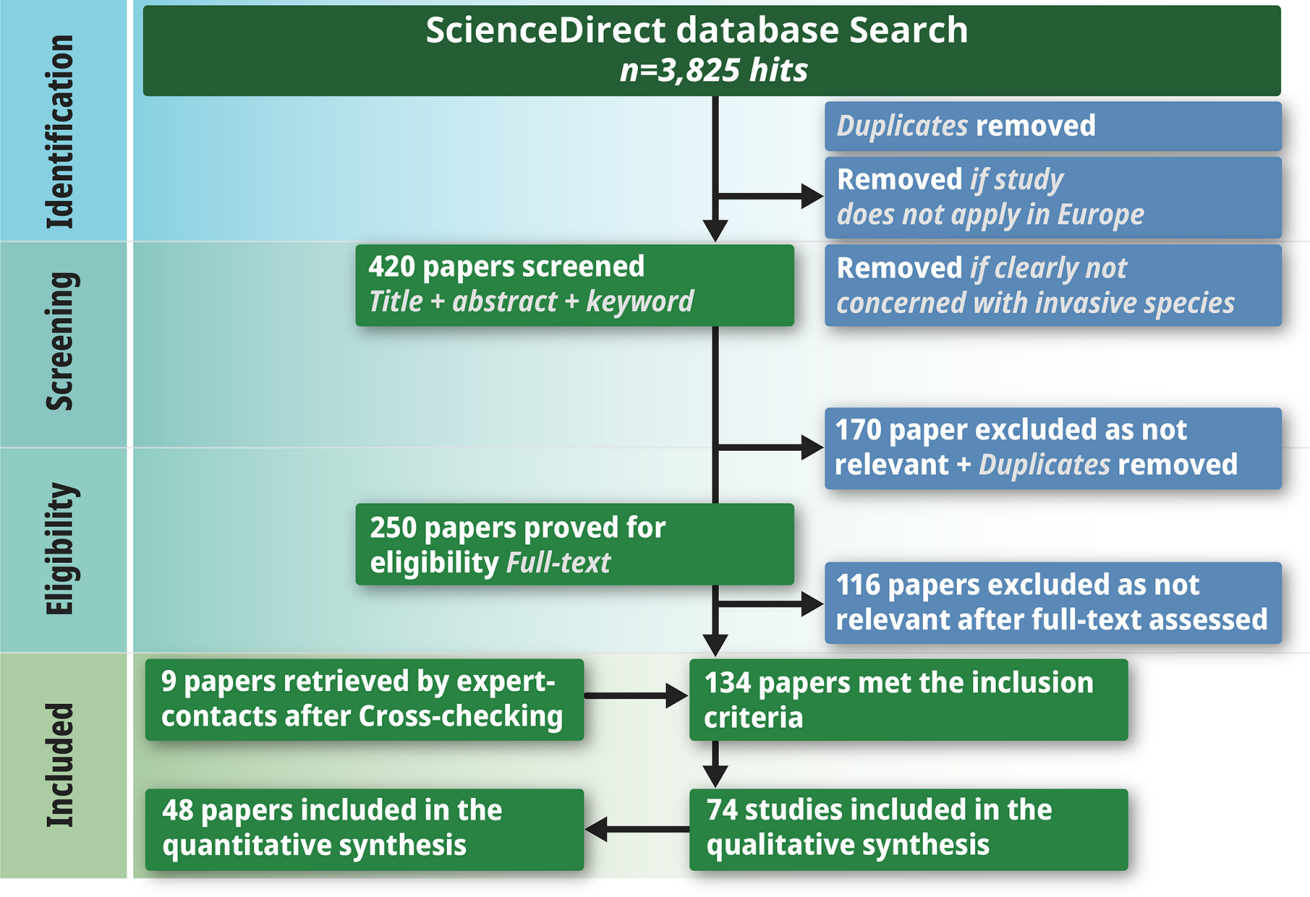
PRISMA diagram outlining the selection procedure for papers on IAS influence on forest regeneration in Central European forests.

**Figure 3:**
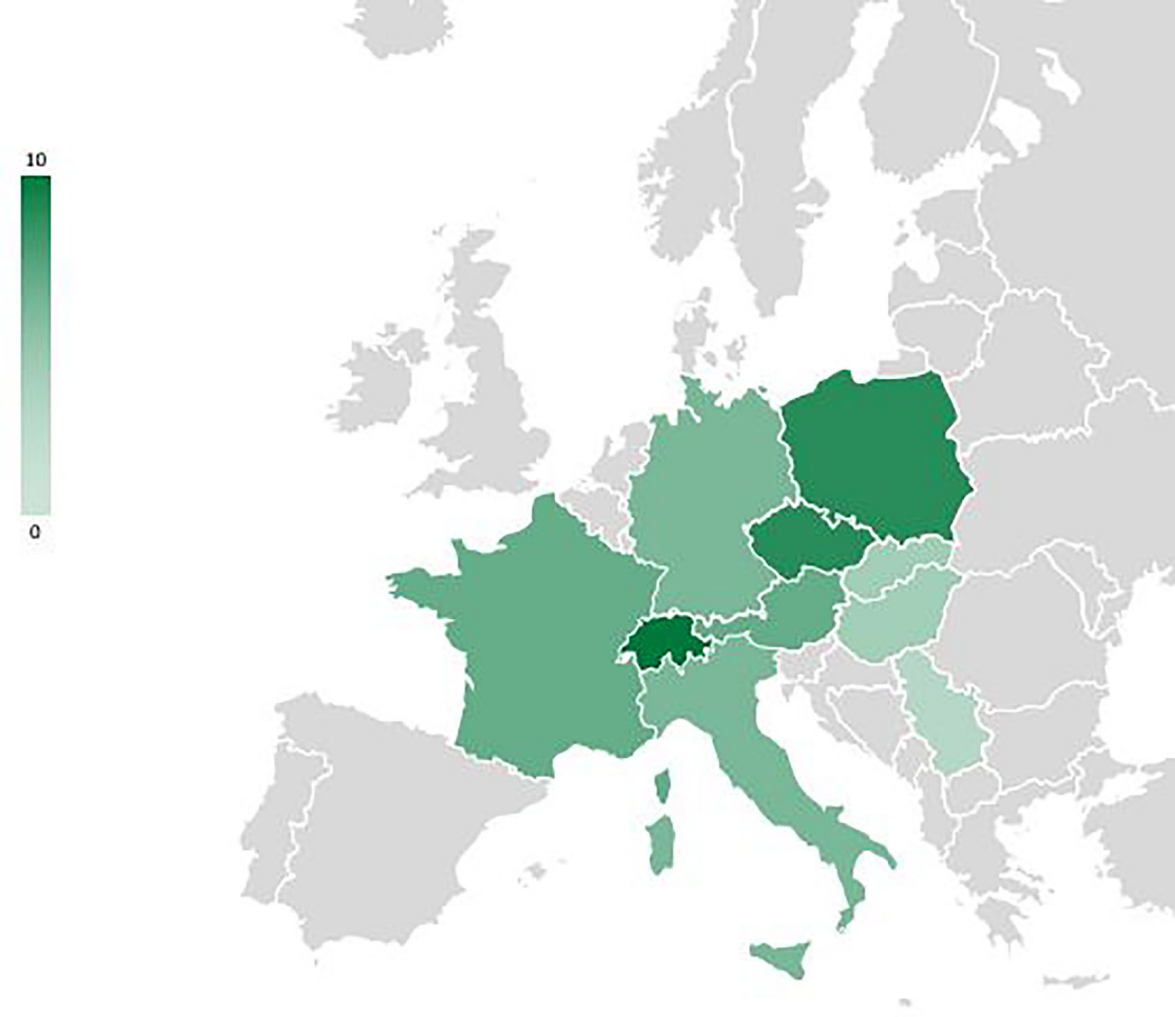
Overview map of Europe showing the countries reported (n=10, in green) in the studies included in the review of IAS impact on regeneration of tree species; scale = number of studies per country.

We followed a multi-step protocol to compile the publications for the literature review (**Figure 2**). Empirical studies fulfilling all of the following criteria were selected for further analysis: 1) published between 1978 and 2019, 2) conducted in European temperate forests as defined in **Figures 1** and **3**) analyzing the direct or indirect impact of alien plant species on regeneration according to the research questions. Reviews, meta-analyzes and book chapters were excluded. In the first step, we focused only on research articles and review articles. The search for publications was performed using the ScienceDirect database (ScienceDirect, 2019). In total, four literature searches were conducted between 7 February and 30 March 2019 using the following search terms:

1. “temperate forest” AND “Europe” AND “invasive plant species” OR “non-native plant species” OR “exotic plant species” ➔ 982 results
2. “regeneration” AND “forest” AND “Europe” AND “invasive plant species” OR “non-native plant species” OR “exotic plant species” ➔ 956 results
3. “biological invasion” AND “vegetation” AND “regeneration” AND “forest” ➔ 224 results
4. “rejuvenation” AND “forest” AND “Europe” AND “invasive plant species” OR “non-native plant species” OR “exotic plant species” ➔ 980 results

In addition, the following five search queries for specific alien plant species, which are among the most frequently mentioned IAS in temperate forests in grey literature, data bases (EPPO, http://gd.eppo.int/; GISD, http://www.iucngisd.org/gisd/; GRIIS, http://griis.org/; 20.02.2019) and local experts were used for cross-checking (Dziub, 2019):

6. “*Solidago gigantea*” AND “forest” AND “Europe” ➔ 63 results
7. “*Impatiens glandulifera*” AND “forest” AND “Europe” ➔ 85 results
8. “*Robinia pseudoacacia*” AND “forest” AND “Europe” AND “invasive” ➔ 246 results
9. “*Fallopia*” AND “forest” AND “Europe” ➔ 160 results
10. “*Ailanthus altissima*” AND “forest” AND “Europe” AND “invasive” ➔ 129 results

These nine search queries produced a total of 3,825 hits. The first reduction removed all hits that did not apply to the selected research area (Central European forests, as stated in **Figure 1**) or were not clearly concerned with invasive species and resulted in 420 remaining publications. For this reduction, only the title, abstract and keywords of each study were analyzed.

In the next step, every duplicated publication was removed, which left 250 publications to be examined more closely. The final selection step was to read the abstracts of these 250 papers to obtain an overview of their contents. Completion of this process left a final number of 134 publications. In addition, we contacted IAS researchers known to members of our research group and asked them to provide lists of their publications during the past years (Dziub, 2019). Cross-checking these lists against the papers we already had resulted in a further nine papers for review. In the end, we had 143 publications to read in full in order to determine whether they provided any answers to our research questions. This reading left us with 74 relevant papers, 26 of which were important in terms of background information but did not deal with our specific research questions. We thus ended up with a grand total of 48 papers relevant for answering our meta-study’s research questions.

### Literature Analysis and Synthesis

The analysis of impact mechanisms followed the definitions by Blackburn et al. (2014), which are in widespread use for socio-economic impact classification of alien taxa (Bacher et al., 2018). The impact mechanisms were slightly adapted for the analysis of reported impacts of alien plant species on tree regeneration. A further goal of this review was to synthesize the management recommendations reported in empirical studies. We collated the technical information and classified it according to IAS management categories, which follow the definitions of the EU Regulation on IAS (EU Regulation, 2014) (**Table 3**): “Early detection”, “Adaptation of silvicultural measures”, “Eradication measures”, and “no management”. The applied taxonomy follows the Plant List (The Plant List, 2013, www.theplantlist.org). Microsoft Excel 2010 was used for data management and data control, and R version 3.4.2 (R Core Team, 2017) was used for processing the data analysis.

## Results

Of the 48 analyzed publications, most studies constitute descriptive empirical research focused on individual forest sites (71%, n=34) or a comparison between two or more forest sites (23%, n=11). Only one study is an experimental study. Four percent (n=2) of the studies do not focus on IAS in forests specifically, but instead on various different land use types (Annex 1).

**Figure 4:**
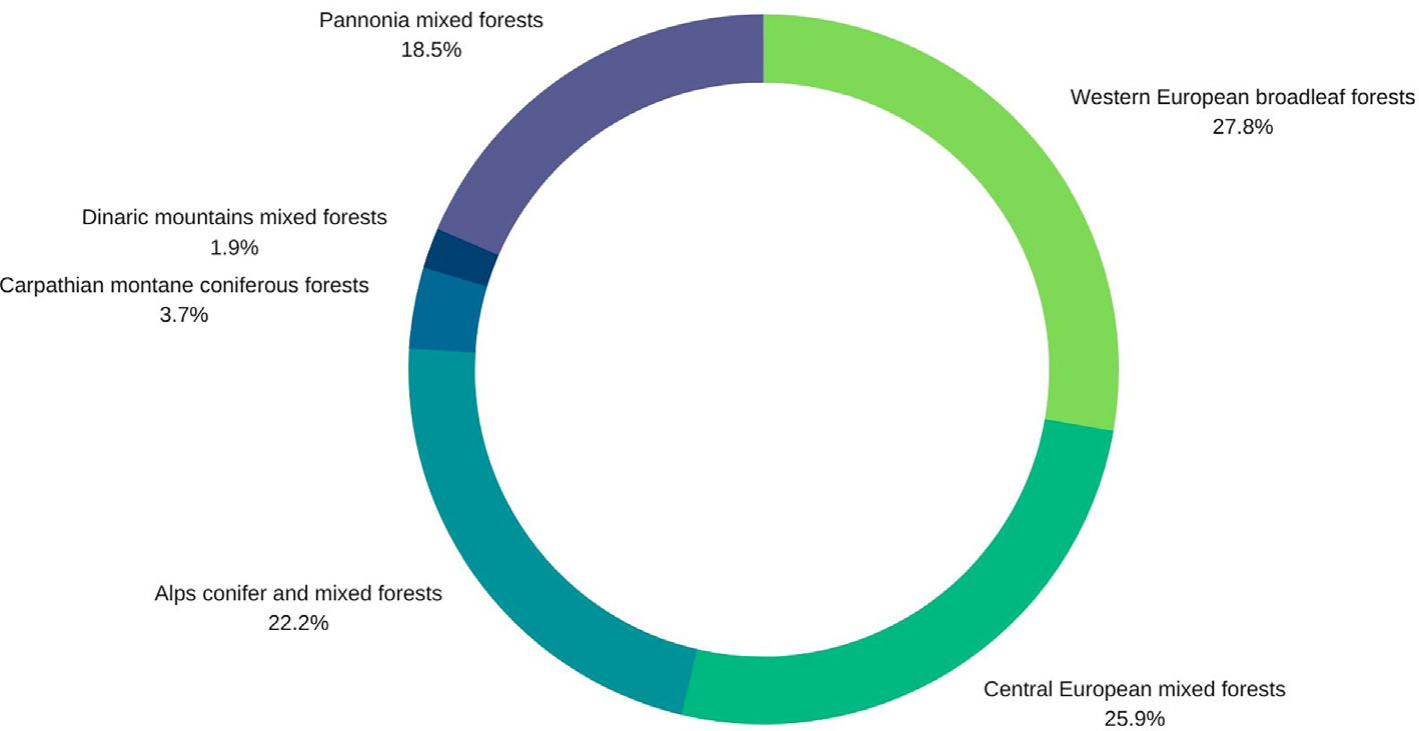
Pie diagram of forest type representation in the 48 papers selected for the quantitative synthesis.

Nineteen percent of the studies are related to unspecified forests at the country level (Alps conifer and mixed forests; Central European mixed forests; Dinaric Mountains mixed forests; Pannonian mixed forests; Western European broadleaf forests), and a further 19% deal specifically with Central European mixed forests or Alps conifer and mixed forests. 25%are specifically on Western European broadleaf forests. Pannonian mixed forests are represented in 15% of the studies, while Carpathian montane coniferous forests appear in only 4%. Studies from Switzerland, Poland and the Czech Republic predominate (**Figure 4**).

The studies are not distributed equally across the study area. Also, out of the entire 41 years considered (1978–2019), only publications from 14 different years within the period from 1994 to 2018 met the criteria for further analysis, and nearly 80% of those studies were published during the past decade (**Table 1**).

**Table 1:** Selected and analyzed studies included in the quantitative synthesis of this meta-study (n=48 papers), showing the number of IAS analyzed in the paper and the country of the research study.

Year	Year and publication	No.of IAS studied	Countries
1994	Schnitzler, 1994	1	FR; GE
1995	Pyšek and Prach (1995)	1	CZ
2005	Grund et al. (2005)	2	CH
	Moravcová et al. (2005)	1	CZ
2007	Chmura and Sierka, 2007	1	PL
	Closset-Kopp et al., 2007	1	FR
	Sebert-Cuvillier et al., 2007	1	FR
2008	Nováková, 2008	12	CZ
2009	Rahmonov, 2009	1	PL
2011	Ammer et al., 2011	1	GE
	Essl et al., 2011	na	AT
	Motard et al., 2011	1	FR
	Pyšek et al. (2011)	na	CZ
2012	Ferréa et al. (2012)	2	IT
	Maringer et al., 2012	15	CH
2013	Petrášová et al., 2013	15	HU; SK
	Radtke et al., 2013	2	IT
	Schmiedel and Tackenberg (2013)	1	GE
	Terwei et al., 2013	2	IT
2014	Halarewicz and Zołnierz, 2014	1	PL
	Höfle et al. (2014)	3	AT
	Kopeć et al., 2014	na	PL
	Rouifed et al. (2014)	1	FR
	Ruckli et al., 2014	1	CH
	Rusterholz et al., 2014	1	CH
	Staska et al., 2014	1	AT
2015	Dyderski et al., 2015	12	PL
	Knüsel et al., 2015	1	CH
	Laube et al., 2015	2	GE
2016	Berg et al. (2016)	10	AT
	Braun et al. (2016)	31	Central Europe
	Nobis et al., 2016	na	PL
	Pěknicová et al. (2016)	3	CZ
	Schiffleithner and Essl, 2016	3	AT; CZ
	Seipel et al. (2016)	2	CH
	Vacchiano et al., 2016	na	IT
2017	Chudomelová et al., 2017	na	CZ
	Florianová and Münzbergová, 2017	1	CZ
	Gaggini et al. (2017)	na	CH
	Nobis et al., 2017	na	PL
	Radovanović et al., 2017	1	RS
	Rusterholz et al., 2017	1	CH
2018	Campagnaro et al., 2018	5	Central Europe
	Erdős et al. (2018)	na	HU
	Gaggini et al., 2018	1	CH
	Łapok et al. (2018)	12	PL
	Rusterholz et al., 2018	1	CH
	Thurm et al. (2018)	na	Europe

The studies were published in between the year 1994 and 2018 (na, no species-specific information stated).

**Figure 5:**
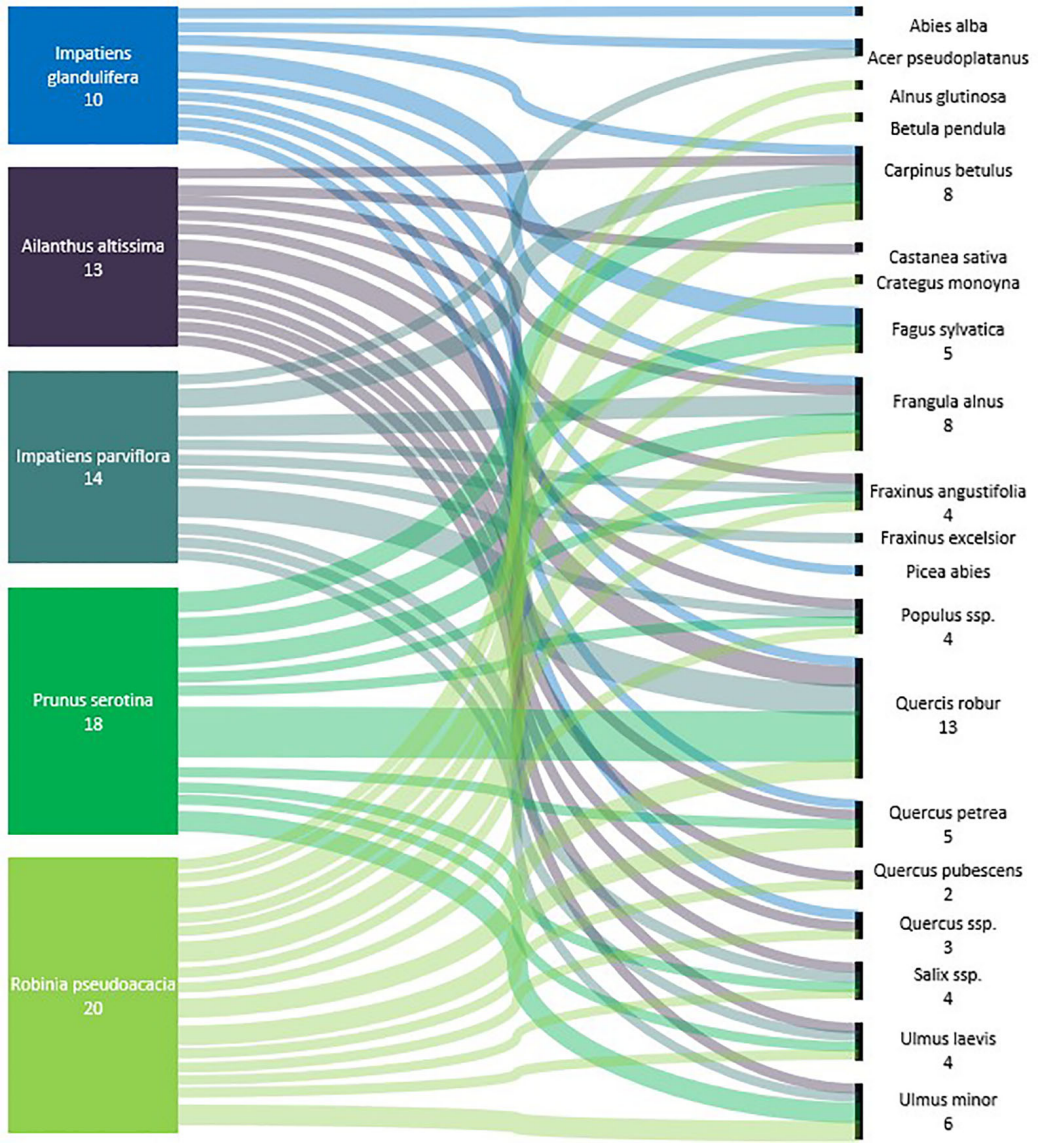
Sankey flow diagram showing the link between the five most frequent IAS with a negative influence on forest regeneration of native tree species in Central European forests (n = number of studies reporting a direct or indirect influence).

In total, 53 alien plant species were targeted by research. The focus of 17 of the relevant studies (35%) lies on providing a list of alien plant species and their effects on forest ecosystems, while 31 studies (65%) analyze or test the impact of specific selected species. Only 28% (n=17) of the studies do not describe at least one impact mechanism of alien plant species on regeneration (**Table 2**). Competition is the most frequently reported impact mechanism (34%, n=21), observed for 30 alien plant species. The alien plant species *Robinia pseudoacacia L*. (9.7%, n=14), *Prunus serotina Ehrh*. (8.3%, n=12), *Ailanthus altissima (Mill.) Swingle* (6.9%, n=10), *Impatiens glandulifera Royle* (4.9%, n=7), *Acer negundo L*. (4.9%, n=7), *Solidago canadensis L*. (4.9%, n=7), and *Impatiens parviflora DC*. (4.2%, n=6) are the most frequently studied species with an impact on forest regeneration in the analyzed publications. In total, 21 tree species are reported to be impacted by IAS in 24 studies. The species whose regeneration is most often reported to be influenced are the temperate tree species *Quercus robur* (n=7), *Fagus sylvatica* (n=5), and *Carpinus betulus* (n=4) (**Figure 5**).

**Table 2:** Impact mechanisms (Competition for resources, Chemical impact on regeneration, Physical impact on regeneration, Structural impact on regeneration, and Indirect impacts through interaction with other species*)* exhibited by the IAS listed in the studies, showing the number of studies and the number of IAS for early detection, adaptation of silvicultural measures, and eradication measures.

	Competition	Chemical	Physical	Structural	Indirect
**No. of studies**	30	4	2	5	8
*Acer negundo L*.	✔	✔	–	✔	✔
*Aesculus hippocastanum L*.	–	–	–	–	–
*Ailanthus altissima (Mill.) Swingle*	✔	–	✔	✔	✔
*Ambrosia artemisifolia L*.	✔	–	–	–	–
*Amorpha fruticosa L*.	–	–	–	–	✔
*Bidens frondosa L*.	✔	–	–	–	–
*Buddleja davidii Franch*.	✔	–	–	–	–
*Catalpa bignonioides Walter*	–	–	–	–	–
*Celtis orientalis L*.	✔	–	–	–	–
*Cinnamomum camphora (L.) J. Presl*	✔	–	–	–	–
*Conzya canadensis (L.) Cronquist*	✔	–	–	–	–
*Diospyros lotus L*.	✔	–	–	–	–
*Erigeron annuus (L.) Desf*.	✔	–	–	–	–
*Fraxinus pennsylvanica Marshall*	✔	–	–	–	–
*Galeobdolon argenatum (L.) L*.	✔	–	–	–	–
*Impatiens glandulifera Royle*	✔	✔	–	✔	✔
*Impatiens parviflora DC*.	✔	–	–	–	–
*Phytolacca americana L*.	✔	–	–	–	–
*Prunus laurocerasus L*.	✔	–	–	✔	✔
*Prunus serotina Ehrh*.	✔	✔	✔	✔	✔
*Quercus rubra L*.	✔	–	–	✔	✔
*Reynoutria japinica (Houtt.) Ronse Decr*.	✔	–	–	–	–
*Reynoutria* ssp.	✔	–	–	–	–
*Robinia pseudoacacia L*.	✔	✔	–	✔	✔
*Solanum chenopodioides Lam*.	✔	–	–	–	–
*Solidago canadensis L*.	✔	✔	–	–	–
*Solidago gigantea L*.	✔	–	–	–	–
*Symphyotrichum lanceolatum Willd*.	✔	–	–	–	–
*Symphyotrichum novi-belgii (L.) G.L.Nesom*	✔	–	–	–	–
*Trachycarpus fortunei (Hook.) H.Wendl*.	✔	–	–	–	–
*Vitis vulpina L*.	✔	–	–	–	–

More than half of the studies (54%, n=26) do not recommend specific management measures for regeneration. In 18% of the studies, adaptation of silvicultural measures is discussed in a species-specific fashion, and 21% of the publications recommend eradication measures for a total of 11 alien plant species. None of the studies specifically recommend not managing alien plants at all (**Table 3**).

**Table 3:** List of IAS with recommended management measures in the studies, showing the number of studies and the number of IAS for early detection, adaptation of silvicultural measures and eradication measures.

	Early detection	Adaptation of silvicultural measures	Eradication measures
**No. of studies**	6	**11**	**10**
*Acer negundo L*.	–	–	✔
*Aesculus hippocastanum L*.	–	–	✔
*Ailanthus altissima (Mill.) Swingle*	✔	–	✔
*Bidens bipinnata L. 1753*	–	✔	–
*Buddleja davidii Franch*.	–	✔	–
*Cinnamomum camphora (L.) J. Presl*	–	✔	–
*Conzya canadensis (L.) Cronquist*	–	✔	–
*Diospyros lotus L*.	–	✔	–
*Heracleum mantegazzianum Sommier & Levier*	–	–	✔
*Impatiens glandulifera Royle*	✔	✔	✔
*Impatiens parviflora DC*.	✔	✔	✔
*Ligustrum sinense Lour*.	–	✔	–
*Lonicera japonica Thunb*.	–	✔	–
*Paulownia tomentosa (Thunb.) Steud*.	–	✔	–
*Phytolacca americana L*.	–	✔	–
*Prunus laurocerasus L*.	–	✔	–
*Prunus serotina Ehrh*.	✔	–	✔
*Quercus rubra L*.	–	✔	✔
*Reynoutria ×bohemica (Chrtek & Chrtková) J.P.Bailey*	–	✔	✔
*Reynoutria japinica (Houtt.) Ronse Decr*.	–	✔	–
*Reynoutria sachalinensis (F.Schmidt ex Maxim.) Nakai*	–	✔	–
*Reynoutria* ssp.	–	✔	✔
*Robinia pseudoacacia L*.	–	✔	✔
*Solanum chenopodioides Lam*.	–	✔	–
*Trachycarpus fortunei (Hook.) H.Wendl*.	–	✔	–

## Synthesis

### IAS With a Negative Influence on Forest Regeneration of Native Tree Species

The detected 53 invasive alien plant species negatively influencing forest regeneration of 21 native tree species, include mostly woody alien tree and shrub species, followed by perennial alien plant species (Annex 1). For most native tree species, the link to the influencing IAS relayed on one or two study only. The highest number of studies was found for the forests regeneration of pedunculate oak (*Quercus robur*), which was reported to be negatively influenced of the most number of IAS, such as *Prunus serotina, Solidago canadensis, Solidago gigantea, Quercus rubra, Impatiens parviflora*, *Robinia pseudoacacia*, and *Reynoutria* sp. (Closset-Kopp et al., 2007; Sebert-Cuvillier et al., 2007; Nováková, 2008; Petrášová et al., 2013; Terwei et al., 2013; Schiffleithner and Essl, 2016; Florianová and Münzbergová, 2017). Similar IAS, are affecting the regeneration of the sessile oak *(Quercus petraea)* and the common hornbeam (*Carpinus betulus)* (Sebert-Cuvillier et al., 2007; Radtke et al., 2013; Schiffleithner and Essl, 2016). The regeneration of Scots pine (*Pinus sylvestris*) was negatively influenced by *Prunus serotina* and *Robinia pseudoacacia* (Sebert-Cuvillier et al., 2007; Rahmonov, 2009).

### Impact Mechanisms

#### Competition for Resources

Competition—defined as the process of alien plants competing with native tree species for resources—is the most commonly reported impact mechanism of alien plant species on the regeneration of tree species. According to the literature analysis, competition for light has the greatest impact on tree seedling establishment (Radtke et al., 2013; Terwei et al., 2013). Light availability is often expressed in terms of canopy cover and the invasion by light-demanding species is likened to management-related disturbances that lead to changes in light availability (Schnitzler, 1994; Terwei et al., 2013; Vacchiano et al., 2016). Under low light availability circumstances, competition for light becomes crucial for the successful establishment of tree seedlings (Closset-Kopp et al., 2007). Terwein et al. (2013) showed that the abundance of *Carpinus betulus* seedlings depends on low herb layer cover, which indicates less competition for light. The canopy gap ecology in relation to competition for light becomes particularly critical in regard to the alien plant species *Prunus serotina* (Closset-Kopp et al., 2007; Sebert-Cuvillier et al., 2007; Terwei et al., 2013; Dyderski et al., 2015; Dreiss et al., 2016). Competition for light of the IAS *Sodiago gigantea, Impatiens glandulifera* and *Conzya canadensis*, was in particular observed in nutrient-rich forests and frequently disturbed sites, such as the riparian mixed oak–elm–ash hardwood forests along great rivers (Fraxino-Ulmetum and Ulmeto-Quercetum) (Petrášová et al., 2013).

Competition mechanisms also include resistance to drought events. Knüsel et al. (2015), for example, showed that *Ailanthus altissima* successfully competes with the tree species *Castanea sativa* due to its drought resistance, which *A. altissima* maintains throughout all regeneration phases.

#### Chemical Impact on Regeneration

Chemical impact on regeneration refers to changes caused by the alien plant to the chemical biotope characteristics of the native environment, nutrient and/or water cycles, disturbance regimes or natural succession. The indirect chemical impact of legume plant species, which have the ability to fix nitrogen from the air, leads to structural ecosystem changes and the creation of new plant communities and therefore negatively affects the forest regeneration.

Few among the examined studies deal with the release of allelochemicals, which impact the regeneration of neighboring tree seedlings directly (Schnitzler, 1994; Rahmonov, 2009; Rusterholz et al., 2018). Chemical impacts also include the ability of alien plants to alter chemical and biochemical soil properties, which in turn can cause changes in the native species richness above and below the ground (Rusterholz et al., 2018). Gaggini et al. (2018) found that the alien species *Impatiens glandulifera* changes the soil fungal and bacterial communities in conifer and mixed forests by releasing naphthoquinones. Another aspect of chemical impacts is the fact that the chemical composition of plant litter from alien plants such as *Robinia pseudoacacia* can cause high levels of nitrogen in the upper soil horizons, thereby exerting an effect on regeneration (Rahmonov, 2009).

#### Physical Impact on Regeneration

Physical impact on regeneration refers to changes to the physical biotope characteristics of the native environment, nutrient and/or water cycles, disturbance regimes, or natural succession. Changes in soil humidity due to invasion by alien plants are among the main effects in this category. This impact is described, for example, for the alien shrubs *Prunus laurocerasus* (Rusterholz et al., 2018) and *Prunus serotina* (Terwei et al., 2013). The physical impact observed of *P. serotina* and *Ailanthus altissima* on co-occuring tree species in temperate forests is linked to the demographic process through which individuals decrease in size and delay mortality (Closset-Kopp et al., 2007; Knüsel et al., 2015).

#### Structural Impact on Regeneration

Structural impact on regeneration refers to alien plants causing changes in the structural biotope characteristics of the native environment, nutrient and/or water cycles, disturbance regimes, or natural succession. At species level the structural impact was observed for the IAS *Prunus serotina, Robinia pseudoacacia* and *Ailathus altissima* (Rahmonov, 2009; Campagnaro et al., 2018). This impact mechanism is mainly discussed as the indirect ability of individual plant invaders to serve as indicators for changes in forest habitat structures and functions as well as regeneration (Campagnaro et al., 2018). Structural impacts on regeneration include the homogenization of species composition in the herb layer, changes to the seed bank, effects on species diversity and changes in the forest structure due to ruderalization (Rahmonov, 2009; Halarewicz and Zołnierz, 2014; Staska et al., 2014; Nobis et al., 2016; Campagnaro et al., 2018). The effects of structural changes to forest ecosystems are time-dependent. For example, structural changes caused by the invasion of a deciduous forest by *Impatiens glandulifera* are documented as becoming visible 5 years after the invasion and causing significant alterations after 13 years (Rusterholz et al., 2017). Oak forests and riparian forests in particular are reported as being affected by the impact mechanism of structural changes caused by alien plants (Kopeć et al., 2014; Nobis et al., 2016; Chudomelová et al., 2017; Nobis et al., 2017; Radovanović et al., 2017).

#### Indirect Impacts Through Interaction With Other Species

This mechanism refers to alien plants interacting with other native or alien taxa (e.g. through any mechanism including pollination, seed dispersal, apparent competition and mesopredator release). Such indirect impacts have been found e.g. for *Impatiens glandulifera* in regard to the arbuscular mycorrhiza symbiosis with a negative impact on the regeneration of *Acer pseudoplatanus* (Ruckli et al., 2014). Furthermore, microarthropod species richness can be affected by *I. glandulifera* invasion in terms of changes to the nutrient cycle, which may indirectly impact regeneration (Rusterholz et al., 2014). Apparent indirect competition however was observed for *Amorpha fructicosa* in poplar and willow habitat and for *Ailanthus altissima* and *Robinia pseudoacacia* in conifer and mixed forests (Radtke et al., 2013; Radovanović et al., 2017). However, detailed studies analyzing the complexity of the indirect impact mechanisms of IAS in ecological network of European forest ecosystems are rare.

#### Other Impact Mechanisms

Hybridization of alien plant species with native tree species as well as the impact mechanisms of disease transmission from alien plant species to native tree species and parasitism of native tree species by alien taxa, which can cause direct or indirect deleterious effects on native tree species, are not relevant for the regeneration of tree species. These impact mechanisms were not described in the 48 analyzed papers.

#### Combined Impact Mechanisms

Most alien plant species seem to exhibit a combination of impact mechanisms affecting the regeneration of native plant species (**Table 2**). Especially, many tree species, such as *Acer negundo, Ailanthus altissima, Prunus laurocerasus, Prunus serotina*, and *Robinia pseudoacacia* have reportedly several mechanisms to impact forest regeration*. Robinia pseudoacacia* originating from North America is a good example of an invading plant species that uses different impact mechanisms at different stages of invasion. It increases nitrogen availability, changes light conditions, creates plant communities and is also associated with allopathic activity (Rahmonov, 2009; Campagnaro et al., 2018). *R. pseudoacacia* can be a desirable and beneficial species for forest management on degraded, sandy, urban, and initial soil, while other studies report its negative impacts on riparian forests, Pannonian mixed forests and Western European broadleaf forests. Its effects particularly affect the natural regeneration of *Ulmus laevis, Ulmus minor, Quercus pubescens, Quercus petrea, Quercus robur, Populus* sp.*, Crataegus monogyna, Betula pendula*, *Pinus sylvestris*, and *Fraxinus angustifolia* (Rahmonov, 2009; Maringer et al., 2012; Petrášová et al., 2013; Radtke et al., 2013; Terwei et al., 2013).

### Management Recommendations

#### Early Detection

Measures for early detection include forest maintenance and restoration (Terwei et al., 2013; Nobis et al., 2017) as well as raising the awareness of forest managers and owners for biological invasions (Motard et al., 2011). Early detection of plant invasion is particularly essential for unmanaged sites, forests at low elevations (Laube et al., 2015) and urban forests (Sjöman et al., 2016). Additionally, it is important to adequately inform citizens about the environmental impact of IAS (Kowarik, 2010; Rupp et al., 2017).

#### Adaptation of Silvicultural Measures

Recommendations concerning the adaptation of silvicultural measures include preventive actions such as the recommendation to plant more native tree species. Furthermore, continuous tree cover and longer rotation periods are recommended where appropriate to promote shadier conditions. The slight thinning of forest areas should be avoided to reduce the risk of management disturbances that may function as pathways for the introduction of alien plant species into the forest. Finally, alternative cutting regimes to simple clearcutting, such as selection or shelterwood systems applied in close-to-nature silviculture, should be preferred (Sitzia et al., 2016). When opening dark forest stands that can be colonized by *Phytolacca americana*, it is recommended to open them quickly and intensively and to begin the regular care in time. Neighbouring stands should not be opened. (Rupp et al., 2017).

#### Eradication Measures

Once alien plants are established, a set of technical measures to control them and ensure the regeneration success of native species are recommended. The eradication measures recommended for alien plant species affecting the regeneration of native tree species include the following tasks: biological control, mowing, hand pulling, manual removal, felling, chemical control, grazing, girdling, and general management of the area.

Less concrete measures for individual IAS were also found in the studied literature. Ammer et al. (2011) launched one of the few experiments to observe how *Impatiens glandulifera* affects the natural rejuvenation of birch and the artificial regeration of spruce and fir. They were able to prove statistically that *I. glandulifera* does not limit the growth of rejuvenation; only isolated losses of fir trees could be determined. This is due to the different growth rhythms of *I. glandulifera* and the examined tree species. The height growth of the native trees begins much earlier than that of the annual *I. glandulifera*, which seems to decouple the competition for resources. The study did not investigate whether the sprouting of trees is affected by *I. glandulifera* (Ammer et al., 2011).

When looking through articles from non-scientific journals, we also found some specific management measures, especially eradication measures. An experiment was performed in Baden-Württemberg, Germany in which *Phytolacca americana* was impregnated in order to prevent its natural rejuvenation sprouting. In order to be able to contain *P. americana* with an economical use of labour and costs, measures must be taken before seed banks are established. The most successful measure was the digging up of the entire plant prior to seed collection (Rupp et al., 2017).

## Discussion

### Review and Synthesis of the Literature

This synthesis is based exclusively on the results of 48 empirical studies conducted in European temperate forests, and its general applicability should therefore be tested in future research. Furthermore, forest management was not included in the search queries, and the synthesis of IAS management approaches and measures consist mostly of expert recommendations that have not been tested in field trials and experiments. Nevertheless, we offer a classification reflecting the approaches taken in IAS management over the past three decades. More detailed information may be included in technical reports in various local languages that were not analyzed within this meta-study.

### The Impact of IAS on Regeneration in Temperate Forests

Temperate forests are not resistant to invasion by alien plant species, which can have a negative influence on the forest regeneration of native tree species. The systematic review of 48 studies determined 53 vascular plant species having a negative influence on forest regeneration. The effect however was not reported evenly for all forest types in Central Europe (**Figure 4**). Riparian forests for instance accounted for the largest portion of studies within our review (25% of all studies) as well as for the highest numbers of IAS. This is in line with Medvecká et al. (2018) who also report the highest percentage of non-native species in riparian forests and highlight that IAS are well-adapted to riparian forests. Their success may be reasoned by occurrence of frequent disturbances and nutrient enrichment due to periodic flooding events in riparian forests (e.g. Kalusová et al., 2013; Medvecká et al., 2018). Natural or anthropogenic disturbances and forest fragmentation favor the spread of IAS in forest ecosystems, by increasing light and nutrient availability (Brothers and Spingarn, 1992; Alston and Richardson, 2006; Raghubanshi and Tripathi, 2009; González-Moreno et al., 2013). However, Martin et al. (2009) determined that anthropogenic rather than natural disturbances drive the rate of invasion.

Such disturbances together with human-induced propagule input support invasions especially along roadsides, railways (Becker et al., 2005) or urban areas (Sjöman et al., 2016) and make them the highly invaded sites even in unfavored landscapes (Kalusová et al., 2013). According to the study by Kalusová et al. (2013), 47 different IAS species are documented in disturbed European forest and woodlands. Alien plant species are capable of altering species composition and species richness of the ground vegetation. Understanding the processes affecting invasion dynamics is crucial for promoting the successful regeneration of native tree species. The complexity of alien plant invasion processes in temperate forests is driven by the invasibility after disturbances of the individual forest ecosystem and the species-specific characteristics of the involved native and non-native species. Most studied species exhibit particular traits that are linked to habitat-independent invaders of disturbed and nutrient-rich areas, such as large seed numbers, anemochore seed dispersal, light demand, and rapid growth rates (Dreiss and Volin, 2013; Schmiedel et al., 2013; Medvecká et al., 2018). Relatively few alien plant species with long life spans and slow growth rates that can form a persistent seed bank and have life histories similar to those of native plant species have been studied. The primary focus of the research examined for our synthesis lies on alien plants that rapidly invade canopy gaps rather than on “slower” invaders of closed canopy forests that might compete with native tree species in their later life stages. Closset-Kopp et al. (2007) conclude that the invasion success of alien plant species, e.g. of *Prunus serotina*, in closed canopy forests is supported by the ability of those IAS to follow a “sit and wait strategy”. Accordingly, alien species following this strategy can invest quickly into growth and reproduction at a favorable moment after a long time waiting for a suitable canopy gap to open up. The “sit and wait” strategy may also support the success of other invasive alien tree and shrub species such as *Ailanthus altissima, Acer platanoides, Robinia pseudoacacia* and *Quercus rubra* in Europe, Asia and North America (Knapp and Canham, 2000; Sanford et al., 2003; Lee et al., 2004). According to Ewald (2009), no annual IAS apart from *Impatiens* spp. has the potential to establish itself in closed forests due to its shadow tolerance. We conclude that processes affecting the invasion of temperate forests with closed canopies are as yet poorly understood and should be studied in more detail given the changing forest management practices. The recent trend toward continuous cover forestry of temperate forests in Europe supports increased canopy closure (Vera, 2000; Schultze et al., 2014; Kirby and Watkins, 2015), which may require further knowledge on its ecological effects such as biological invasibility.

### Implementation in Forest Management

Invasive alien plants represent an increasingly noticeable problem in the context of the regeneration of native tree species. Various potential management measures to be applied at different stages of biological invasions were identified in our synthesis of 48 studies. The implementation of early detection tools by forest managers and forest owners as well as by the general public can help to identify the spread of alien plants at an early stage as well as supporting the establishment of an early warning system (Kutnar and Pisek, 2013; Sitzia et al., 2016). In particular, species that are recognized invasive in other countries or outside Europe should be treated with caution and prevented where possible (Rejmánek, 2014).

In connection with IAS, management measures in forests are indispensable (Medvecká et al., 2018). Previous studies have shown that a deeper understanding of the driving factors for forest regeneration is needed to assess the impact of IAS in silvicultural scenarios and to improve the silvicultural planning (Montoro Girona et al., 2017; Montoro Girona et al., 2019). Worldwide novel approaches for silvicultural treatments in alternative to clear-cutting have been recently investigated in response to sustainable forest management (Montoro Girona et al., 2016). An adapted management of canopy cover and tree density can be applied to regulate inter-species competition and locally reduce unfavored IAS while promoting native species (Sitzia et al., 2016). Therefore, partial cutting regimes such as shelterwood, selective cutting or seed-tree treatments seem to ensure regeneration and limit the risk of competition with IAS (Montoro Girona et al., 2018).

Furthermore, it is important that a rethinking of forest management takes place. Many IAS would actually have no chance to establish themselves in forests due to their plant sociological characteristics (Abts, 2004) and the implementation of appropriate silvicultural systems would prevent their spread (Sitzia et al., 2016). That they are able to take hold and spread anyway is frequently owed to the destruction of natural biotopes and the disturbance of natural ecosystem dynamics offering a plant-sociological advantage to the IAS (Abts, 2004; Martin et al., 2009; Kowarik, 2010). In forests, forest roads and wood storage areas in particular represent potential settlement areas for IAS (Becker et al., 2005; Haseke and Remschak, 2010).

## Conclusion and Future Research

Alien plant invasions impact forest regeneration directly and indirectly. The 48 studies included in our synthesis discuss a total of 53 invasive alien plant species existing in European temperate forests. We identified five impact mechanisms through which alien plant species affect the regeneration success of native tree species: competition, chemical impacts, physical impacts, structural impacts, and indirect impacts through interaction with other species. Among these impact mechanisms, competition is by far the best-analyzed. Our synthesis shows that physical and chemical impacts as well as changes in soil properties can have significant effects on native species composition and thus on natural regeneration. Further species-specific studies would be very useful in this context to attain a better understanding of ecosystem processes, but also of the influence of forest management purposes. In general, more research is needed to achieve a better understanding of the effects of forest management measures.

Almost no information on the actual effectiveness of management measures is provided in the studies, and we therefore conclude that science and practical forest management need to be linked better and more closely. Long-term experiments developed together with forest managers and owners may help to improve the translation of research results into practice. The review results also show that we lack species-specific experiment designs to investigate the effects of impact mechanisms or management measures in detail.

Knowledge on the possible impacts of alien species is crucial for decision-making processes. For example, forest managers may ask whether natural regeneration is less likely to succeed without further investments into eradication measures when alien plants such as *Impatiens glandulifera* occur on their managed sites. Invasive plants species are increasingly representing a problem in terms of management of the regeneration of native tree species. In the context of climate change and the expected increase in disturbances in forest canopies, studies on demographic traits and gap dynamics will become more important in order to identify the early stages of biological invasions by species following the “sit and wait” strategy. Oftentimes, the spread of IAS is promoted by the destruction of natural biotopes resulting in plant-sociological disadvantages for the regeneration of native trees.

## Data Availability Statement

The raw data supporting the conclusions of this article will be made available by the authors, without undue reservation, to any qualified researcher.

## Author Contributions

ML and KL conceived of the presented idea. ML and KL developed the theory and performed the computations.

## Conflict of Interest

The authors declare that the research was conducted in the absence of any commercial or financial relationships that could be construed as a potential conflict of interest.
